# The System of “Concours”

**Published:** 1847-10

**Authors:** 


### PART V.—EDITORIAL.

### ARTICLE I.

### THE SYSTEM OF "CONCOURS."

A paragraph is going the rounds of the Medical Journals, quoted from the London Medical Gazette, to the effect that the "Chamber of Peers has come to a vote by which the system of Concours is abolished in France."

Not having before us any authentic account of this action we of course are not prepared to confirm or dispute the statement, but admitting its correctness there is no good reason to apprehend that the popular branch of the government will concur and vote its discontinuance.

We might express our surprise that independent journalists in this country should be found to applaud such a retrograde movement of a legislative body, constituted like the French Chamber of Peers. The "laws of September" against the liberty of the press, of speech, and of thought, the incarceration of editors, and liberal writers, and similar acts which it would be out of place to enumerate here, have been for seventeen years the principal work of that servile branch of a corrupt government. But after all the restraints imposed upon men of independent minds there still remained a career by which they might without government patronage, and often in opposition to government candidates, rise to eminence. It was by the public trials in all the different departments of the University, and thus interfering with this in any degree, was an act worthy to follow the antecedents of such a Chamber. If something liberal had been adopted in its stead there might be some excuse, but as it restores all the appointments to the ministry, and places them upon the same footing as others of a political nature, it is stange indeed that it should find advocates with us.

The editor of the Buffalo Medical Journal in commenting upon the vote above alluded too, states some objections to the system, which are founded in a great measure upon a misapprehension of its true character.

He seems to think it supercedes all other evidence of merit; whereas it is used in addition to those at present employed in this country. He objects that the judges might be subject to improper influences, but the same objection applies to a trial by jury. No one supposes that while men are the judges, theoretical perfection in a system can be attained, but this gives what we have not at present, a fair trial before impartial judges in public, and guarantees a good if not the best selection.

He supposes that an incumbent thus selected must hold the place for life, but this would not necessarily follow if he should neglect his duties.

This system has been attended with the most brilliant and satisfactory results. It has gathered at Paris the talent of many countries. Orfila from Spain, Dumas from Switzerland, and Ricord from America, are but specimens of what it has done for France. A mere enumeration of the members of the faculty of the School of Medicine would be a sufficient proof of its excellence. There is an objection to its introduction which we confess to have scarely expected to meet with. It is in substance, that there is no occasion for it, the present state of things being good enough.

We have no disposition to undervalue the qualifications of the professors in our medical schools, but under the present system they have not all the advantages they might possess for arriving at the highest degree of excellence. They commence teaching for the most part late in life, without experience in speaking or any studies directed especially to qualify them for the task of instruction.

Look for a moment at our medical literature. Are not nearly all the books published foreign, with notes by the "American editor"? Are not a large proportion of the medical journals circulated among us from Europe? Can they describe correctly the diseases of this country? And what a state of the profession do these facts show?

The call for the reform has been heard from every quarter of the country, and it is worthy of being remarked, that it came from the societies and not from the schools, and that these latter are regarded as to a certain extent as separated from the mass of the profession. This feeling will grow unless medical institutions are greatly reformed.

Having had an opportunity of witnessing personally the operation of the system of concours, it appeared to us admirable and peculiarly adapted to the character and spirit of our institutions, and if for political reasons it should be discontinued in France it would only be necessary for the leading schools in this country to adopt it, and students will soon flock to them from every country, instead of as at present seeking instruction abroad.

Since writing the above we find the following in the Annalist which will give more light upon this interesting subject.

"*To the Editor of the Annalist, Sir*:—In a very recent number of your journal, I noticed, with much surprise, an article headed "The Concours abolished in France," in which it is positively asserted that this institution—regarded in France as the bulwark of safety to the profession—"is now formally, and as may be fairly concluded, "forever abolished;" and, as I happen to be "among the number of your brethren who have lauded this method of filling vacancies, &c.," and hope yet to see it generally introduced into this country, I ask leave to present a few *facts* in connection with the subject, in order to counteract the erroneous impression that your article is calculated to convey, of the method having been tried abroad and abandoned, as imperfect and insufficient.

"The truth is that the Concours *has not been abolished in France;* and in so far as I am informed on the subject, no attempt has been made to interfere with it except in the case of appointments to full Professorships. When the new Medical Bill was introduced into the Chamber of Peers, it was proposed that in lieu of the Concours, appointments to professorships in the principal Medical Colleges should be made "by presentation," as it is called;

*i. e.*, the names of a certain number of persons should be presented by various scientific bodies, (the faculty in which the vacancy occurred, the Academy of Sciences, and the Royal Academy of Medicine,) to the government, and the selection and appointment were to be made by the Minister of Public Instruction. This was a government modification of the bill, and intended, it is asserted, to secure additional patronage. Previously to introducing the amendment, the Minister requested the opinion of the faculties of Paris, Montpellier and Strasbourg, the only bodies in France empowered to grant medical degrees, and they all objected in the strongest terms to the proposed change. The vote on the question in the Paris faculty stood thus: *against the proposition:* MM. Orfila, Marjolin, Richard, Piorry, Bouillard, Bérard, Gerdy, Blandin, Roux, Velpeau, Denouvilliers, Dumeril, and Cloquet—13; *in favor of the change:* MM. Andral, Chomel, and Fouquier—only 3—and as two of these are attaehcd to the royal family, and the third receivcd his present chair through the Minister, and not by the Concours, it might naturally be expected that they would favor the views of the government.* Thus the vote may be considered unanimous in favor of maintaining the existing order of things in relation to this subject.

"Notwithstanding this opposition of the Professors themselves, and almost the whole body of the medical press, the minister adhered to his determination; and, supported by MM. Cousin and Thénard, the bill passed through the Chamber of Peers. It has not yet been acted upon, however, by the Chamber of Deputies; and as that body is less directly under the influence of the government, and numbers among its members, Monsieur Malgaigne, and other distinguished medical men, known to be advocates of the Concours, it remains to be seen whether this objectionable and unpopular feature will not be removed from the bill before it is allowed to become a law. Should it pass, however, it will, as I have already stated, apply only to the cases of full Professors, for the very first article of the bill contains all the directions required for carrying out the system of concours in other cases; and all adjunct Professors, Hospital Surgeons, Externes, Internes, and Apothecaries to the Hospitals, will continue to be chosen as heretofore.

"Thus you will perceive that the Concours, instead of being abandoned in France, is, after a long trial, fully recog-

*M. Fouquier. is first Physician to the King; M. Chomel Physician to the Duchess of Orleans; and M. Andral did not concour, I am informed, for the place which he now occupies.

nized—in a reform medical bill too—as being the best test of merit, and admitted to be applicable to all appointments except those the patronage of which it may be desirable for a government to retain.

"My sole object in troubling you with the request that you will cause this letter to be inserted in an early number of your journal, is to correct the error into which you, in common with the Editor of the *Medical Examiner*, and other medical journals, have inadvertently fallen; in consequence, perhaps, of a false assertion in the *London Medical Gazette*, (quoted by the *Examiner*,) which journal appears, in this instance at least, to be rather more anxious to abuse the "French system," that to adhere to truth in its statements.

"In a communication like the present, intended solely to correct an error of fact, I do not judge it expedient to enter into a discussion of the merits of the various plans pursued for filling vacancies in Medical Colleges; still, as an assertion is made in your article to the effect that the plan of electing *Lecturers* for a year possessess "many of the advantages of the Concours," and "is entirely free from its objectionable features," I cannot refrain from asking to be permitted to differ with your entirely on this question; and to object to the method on the ground that *without* its advantages, it embraces, what I conceive to be one of the very few imperfect features of the system which you condemn.

"With sentiments of esteem and regard, I remain

Respectfully yours,

F. Campbell Stewart.

"640 *Broadway*, *October* 7, 1847."

